# The Physiological Mechanisms of Transcranial Direct Current Stimulation to Enhance Motor Performance: A Narrative Review

**DOI:** 10.3390/biology13100790

**Published:** 2024-10-02

**Authors:** Shuo Qi, Lei Cao, Qingchun Wang, Yin Sheng, Jinglun Yu, Zhiqiang Liang

**Affiliations:** 1 School of Sport and Health, Shandong Sport University, Jinan 250102, China; qishuo@sdpei.edu.cn (S.Q.);; 2 National Football Academy, Shandong Sport University, Jinan 250102, China; 3 College of Competitive Sports, Shandong Sport University, Jinan 250102, China; 4 School of Exercise and Health Sciences, Xi’an Physical Education University, Xi’an 710068, China; 5 Faculty of Sports Science, Ningbo University, Ningbo 315211, China

**Keywords:** transcranial direct current stimulation, motor performance, mechanism of action

## Abstract

**Simple Summary:**

After outlining the role of tDCS in enhancing physical performance, this study explores its mechanisms of action. The primary focus is on how tDCS improves motor abilities and motor skill by modulating the resting membrane potential of neurons, enhancing synaptic plasticity, and strengthening the functional connectivity of neural networks. tDCS has demonstrated significant potential in improving motor function and facilitating rehabilitation, which is particularly valuable for athletes, the elderly, and patients with neurological injuries. Its broader social impact lies in its accessibility as an economical, convenient, and effective tool that can be widely applied in sports science, rehabilitation medicine, and cognitive neuroscience to enhance individual performance and quality of life.

**Abstract:**

Transcranial direct current stimulation (tDCS) is a non-invasive neuromodulation technique that applies a stable, low-intensity (1–2 mA) direct current to modulate neuronal activity in the cerebral cortex. This technique is effective, simple to operate, affordable, and widely employed across various fields. tDCS has been extensively used in clinical and translational research, with growing applications in military and competitive sports domains. In recent years, the use of tDCS in sports science has garnered significant attention from researchers. Numerous studies have demonstrated that tDCS can enhance muscle strength, explosive power, and aerobic metabolism, reduce fatigue, and improve cognition, thereby serving as a valuable tool for enhancing athletic performance. Additionally, recent research has shed light on the physiological mechanisms underlying tDCS, including its modulation of neuronal resting membrane potential to alter cortical excitability, enhancement of synaptic plasticity to regulate long-term potentiation, modulation of neurovascular coupling to improve regional cerebral blood flow, and improvement of cerebral network functional connectivity, which activates and reinforces specific brain regions. tDCS also enhances the release of excitatory neurotransmitters, further regulating brain function. This article, after outlining the role of tDCS in improving physical performance, delves into its mechanisms of action to provide a deeper understanding of how tDCS enhances athletic performance and offers novel approaches and perspectives for physical performance enhancement.

## 1. Introduction

**Figure 1:**
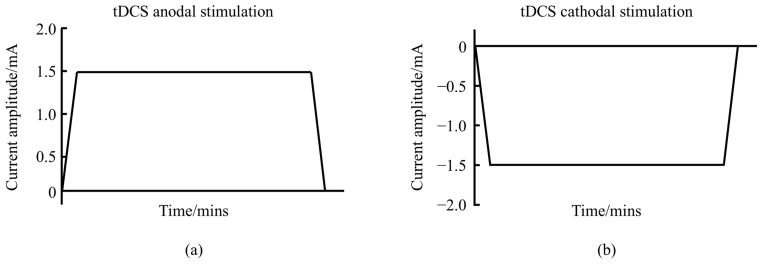
Schematic diagram of the waveform of tDCS. (**a**) represents the anodal tDCS waveform schematic. (**b**) represents the cathodal tDCS waveform schematic.

Transcranial direct current stimulation (tDCS) is a non-invasive brain stimulation (NIBS) technique that uses a steady, low-intensity direct current (1–2 mA) to modulate cortical neuronal activity [1]. The tDCS system consists of a battery-operated stimulator and two electrodes (anode and cathode) placed on the scalp to deliver the current. The most commonly used electrodes measure 5 × 7 cm^2^, although different sizes are sometimes employed [2]. tDCS can be categorized into anodal and cathodal types, with waveform variations shown in Figure 1. The current travels through the scalp, across the outer cortical layers, and reaches the cortex, where it modulates the membrane polarity of neurons in the cerebral cortex [3]. Direct current flows from the anode to the cathode, inducing changes in neuronal electrical activity and thereby altering synaptic efficiency.

Sports performance refers to an individual’s ability to execute a movement or series of movements through optimized motor patterns and efficient motor skills, supported by good physical fitness [4]. It not only impacts athletes’ performance but also influences soldiers’ combat capabilities, the growth and development of children and adolescents, and the health management of the elderly. Furthermore, sports performance reflects the productivity of society as a whole and contributes to a country’s international competitiveness. Multiple factors, including physiological and psychological aspects, influence sports performance. In the realm of competitive sports, there has been a continuous search for effective methods to enhance performance. Recently, non-invasive neuromodulation techniques aimed at improving sports performance have gained significant attention. Among these techniques, transcranial direct current stimulation (tDCS) has been widely studied. Researchers have explored the use of tDCS to improve athletic performance in healthy individuals and athletes, particularly in enhancing muscle strength, delaying fatigue, facilitating motor-skill acquisition, and improving movement sensation [5,6,7,8,9].

In recent years, the journal ***Nature*** has published multiple articles on tDCS-related technologies and their applications. For instance, in 2016, Reardon reported that the U.S. Ski Association partnered with Halo Neuroscience to develop a device using transcranial direct current stimulation (tDCS) technology. This device, integrated into a headset, delivers a mild electrical current to the user’s brain through stimulation electrodes, enhancing motor abilities such as explosive power and flexibility. The headset’s design is based on the principles of tDCS. In 2017, Hornyak described tDCS as a novel “neural priming” tool for improving motor performance, suggesting that it enhances neuromuscular connections, increases neuromuscular recruitment, and improves coordination between internal and external muscles in athletes [10]. With the continued advancement of brain science, tDCS has gained widespread use in clinical research and is increasingly applied in competitive sports [11]. In 2016, the United States Ski and Snowboard Association (USSA) began using tDCS technology to train Winter Olympic ski jumpers, aiming to improve their explosiveness and coordination. Additionally, athletes from the National Football League (NFL), National Basketball Association (NBA), as well as Olympic cycling, triathlon, and golf, have adopted Halo Sport headphones, based on tDCS principles, to enhance their sports performance [12].

Studies in sports science have demonstrated that anodal transcranial direct current stimulation (tDCS) can enhance muscle strength by increasing the excitability of corticospinal tract conduction [13,14]. For instance, 10 min of anodal tDCS has been shown to improve left ankle plantarflexion strength [15], while 20 min of anodal tDCS can increase maximal voluntary contraction force in the wrist extensors, left biceps brachii, shoulder internal and external rotators, and knee extensors [16,17,18]. Muscle strength is crucial for athletes to execute technical movements and achieve high performance during competitions. Anodal tDCS has been found to enhance the muscle strength of adolescent football players, improving their execution of various skills during games [19]. In the general population, anodal tDCS has also been shown to significantly improve muscle strength and physical fitness by increasing relevant muscle strength, load capacity, and the number of maximal strength repetitions [20].

Xiao et al. (2020) found that 20 min of high-precision tDCS targeting the sensorimotor region improved static balance in healthy adults. Balance is a fundamental skill for athletes, especially in non-periodic sports, as it directly influences their physical and technical performance. However, further research is needed to determine whether tDCS can improve both dynamic and static balance in elite athletes [21].

Motor skills, defined as the ability to master and efficiently perform specific movements, depend on synaptic plasticity and functional connectivity across different regions of the cerebral cortex. tDCS has been shown to facilitate motor-skill acquisition and consolidation in healthy individuals [22,23]. Zhu et al. (2015) found that cathodal tDCS stimulation of the left dorsolateral prefrontal cortex inhibited verbal working memory activity, reducing the involvement of episodic verbal analysis in motor control, which improved golf-putting performance in healthy college students [24]. Although tDCS has a wide range of applications in exercise science, the precise neurophysiological mechanisms through which it enhances athletic performance are yet to be fully understood.

Although the exact neurophysiological mechanisms by which tDCS enhances motor performance have not yet been fully elucidated, several prominent theories suggest its effects are primarily mediated through multiple mechanisms. These include modulating neuronal resting membrane potential to alter cortical excitability, enhancing synaptic plasticity to regulate long-term potentiation (LTP)-like effects, improving regional cerebral blood flow (rCBF) via neurovascular coupling, modulating brain network functional connectivity to activate and strengthen specific brain regions, and increasing the content and release of excitatory neurotransmitters.

## 2. Physiological Mechanisms of tDCS Action

### 2.1. Modulating the Resting Membrane Potential of Neurons to Change the Excitability of the Cerebral Cortex

Neurons are the fundamental structural and functional units of neural tissues, and action potentials are initiated when the depolarization of the neuronal resting membrane potential reaches the threshold [25,26]. tDCS can alter the excitability by regulating the resting membrane potential of neurons, and studies have shown that when the primary motor cortex (M1) region of the brain is used as the stimulation region, the anodal tDCS placed above the M1 region modulates the resting membrane potential of neurons, approaches depolarization, and increases the excitability of the cerebral cortex, influencing spinal cord neural pathways, enhancing motor unit recruitment, and ultimately enhancing motor performance [13,14]. Twenty minutes of anodal tDCS intervention significantly increased the maximal voluntary contraction force of the ulnar extensor carpi radialis brevis, biceps brachii, biceps femoris, and rectus femoris muscles in subjects [16,17,18]. However, cathodal tDCS modulates neuronal membrane potentials close to hyperpolarization in healthy subjects and suppresses cortical excitability [26,27]. In addition, tDCS increases the excitability of the M1 region when it acts on this region, which may lead to a sustained neural drive of motor neurons, thus improving connectivity between the M1 area and other regions and improving muscle fatigue [28,29,30]. It has been shown that when anodal tDCS was applied to the M1 region, it improved muscle performance and reduced neuromuscular fatigue in subjects [31,32]. Evidence from human primary motor cortex models and animal models suggests that glutamatergic synapses appear to be at least one of the drivers of DC stimulation-induced plasticity, particularly N-methyl-D-aspartate receptor (NMDAR). Pharmacological studies have shown that a blockade of NMDAR prevents tDCS-induced changes in excitability, both in anodal and cathodal tDCS, whereas NMDAR agonists potentiate the increase in excitability induced by anodal tDCS [33,34].

**Figure 2:**
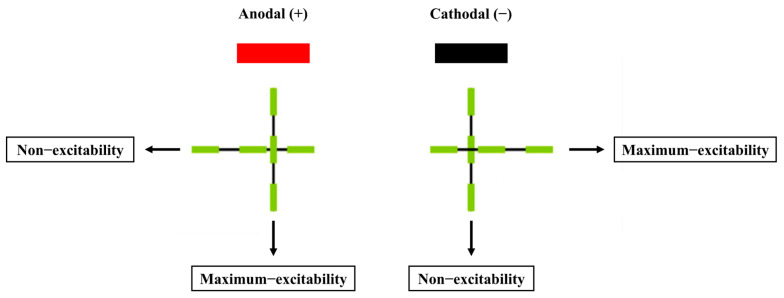
The effect of tDCS on axonal excitability and the relationship between electrode polarity and axonal orientation [36].

However, some studies have shown that cathodal tDCS can also elicit excitatory effects in specific brain regions and improve motor performance. For instance, Ballard et al. (2019) demonstrated that cathodal tDCS applied to the cerebellum facilitated motor-skill acquisition, while anodal tDCS produced the opposite effect, which contrasts with findings from M1 stimulation [35]. Similarly, Zhu et al. (2015) found that cathodal tDCS targeting the left dorsolateral prefrontal cortex (DLPFC) enhanced participants’ golf-putting performance [24]. These discrepancies may be explained by the “axonal modulation” theory [36], which posits that axons are more sensitive to electrical stimulation than dendrites or cell bodies, and the excitability induced by tDCS polarity is dependent on axon orientation. As illustrated in Figure 2, cathodal tDCS excites axonal fibers aligned parallel to the electrode surface and inhibits those perpendicular to it, whereas anodal tDCS produces the opposite effects [36]. Therefore, “axonal modulation” could account for the contrasting outcomes of tDCS applications. In the M1 region, where most pyramidal neurons are vertically oriented, anodal stimulation tends to be more effective.

tDCS can alter cortical excitability and thus enhance the motor performance of the organism. Regarding the polarization mechanism of tDCS, the direction of the current relative to the position of the neurons may play an important role. It is worth noting that the effect of tDCS on neuronal excitability depends on the location of the neuron on which the tDCS is applied, such as the dendrites, intermediate neurons, and long axon terminals, and the spatial location of the neuron or axon and other structures in relation to the electric field. In addition, key elements of the effectiveness of the action of tDCS technology include the specific location of the electrodes, the choice of electrode configuration, the strength of the applied current, the duration of the stimulation, and the frequency and periodicity of the treatment. The electrodes were usually placed in relation to the target brain area to ensure the effectiveness of the stimulation, and common configurations include anode and cathode settings. The current intensity was usually set between 1 and 2 mA and needs to be adjusted according to the study objectives and individual tolerance. The duration of stimulation was typically between 10 and 30 min, while the frequency of treatment depends on the specific purpose of the application, such as cognitive enhancement or motor function recovery. Through the precise integration of these elements, tDCS technology is able to maximize its effectiveness in clinical and research applications.

### 2.2. Enhancement of Synaptic Plasticity and Modulation of LTP-like Effect

The synapse plays a crucial role in integrating neural signaling and facilitating cellular connections, serving as the fundamental unit of neural circuit activity. It plays a key regulatory role in neural communication. Synapses are not static structures but are constantly modified by various stimuli. External stimuli activate neurons across the brain, leading to structural and functional changes at the synapse, known as synaptic plasticity [37,38,39]. Synaptic plasticity can result in either structural or functional reorganization of neurons [40]. The most well-known form of functional plasticity is the LTP-like effect, characterized by persistent synaptic strengthening, a critical mechanism in learning and memory [41]. Since there is a delicate balance between network structure and its relevant functions, we can infer synaptic function through its component parts—axon terminals and dendrite spines [42]. The LTP-like effect is induced by high-frequency stimulation, which engages ligands or ion channels on the neuronal membrane. This effect forms the physiological basis for neural circuit remodeling and the enhancement of brain functions, such as learning and memory [43]. The long-term depression (LTD)-like effect exerts the opposite effect to that of the LTP-like effect. The mechanisms of LTP- and LTD-like effects can be described as follows: some kind of stimulus (anodal tDCS) causes the release of glutamate from the presynaptic membrane, and glutamate binds to alpha-amino-3-hydroxy-5-methyl-4-isoxazolepropionic acid receptor (AMPAR) on the postsynaptic membrane, which opens the receptor channel, and more sodium ions enter the cytosol through the AMPAR channel. For N-methyl-D-aspartic acid receptor (NMDAR), there are magnesium ions “guarding” the gate, but due to the gradual entry of many sodium ions into the cytosol the magnesium ions are washed out, the NMDAR channel opens, more calcium ions enter the cytosol, and calcium ions enter, which opens more AMPARs on the postsynaptic membrane and generates the LTP-like effect [44].

**Figure 3:**
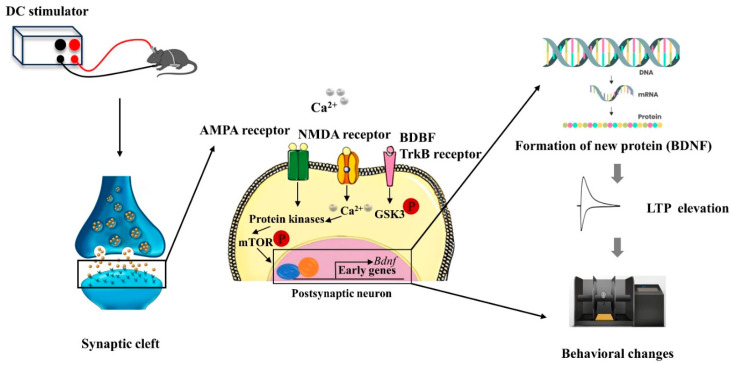
Schematic diagram of the molecular mechanism of tDCS action [43,44].

As illustrated in Figure 3, anodal tDCS stimulation of cortical regions can depolarize the neuronal cell membrane potential, leading to the release of neurotransmitters from the presynaptic membrane. These neurotransmitters bind to NMDAR and AMPAR on the postsynaptic membrane, resulting in an upregulation of intracellular calcium ions in the postsynaptic neuron. This increase in calcium can activate protein kinases, which subsequently promote the production of brain-derived neurotrophic factor (BDNF) by regulating the mechanistic target of rapamycin (mTOR) signaling pathway. As a long-term mechanism, this process enhances gene transcription, leading to the synthesis of BDNF. Ultimately, BDNF contributes to the formation of new proteins that enhance the LTP-like effect and improve behavioral functions [43,44].

Research on the aftereffects induced by tDCS has shown that the effects can persist beyond the duration of the stimulation itself, a phenomenon believed to be linked to cortical synaptic plasticity [45]. When an inhibitor of NMDAR is used, the aftereffects produced by tDCS are attenuated. When an agonist of NMDAR is used, the aftereffects produced by tDCS are enhanced [46]. Furthermore, animal studies indicate that glutamatergic synapses are key drivers of tDCS-induced synaptic plasticity, particularly in relation to NMDAR activity. Pharmacological investigations have demonstrated that blocking NMDAR prevents changes in excitability induced by tDCS, whereas NMDAR agonists facilitate increases in excitability following anodal tDCS. These findings suggest that the duration of tDCS aftereffects is closely tied to synaptic plasticity within the glutamatergic system [34].

Physiologically, dendrites respond to synaptic plasticity stimuli by altering their structural and functional characteristics, which facilitates learning and memory formation. As the postsynaptic sites of neurotransmission, the structural plasticity of dendritic spines significantly impacts the efficiency of synaptic transmission and serves as the basis for the establishment and reorganization of neural circuits during the acquisition of motor skills [47,48]. Research has demonstrated that transcranial direct current stimulation (tDCS) enhances dendritic spine density in a mouse model of middle cerebral artery occlusion, promoting neuroplasticity after a stroke [49,50]. Barbati et al. (2020) found that tDCS enhanced forelimb motor skills in mice, and increased dendritic spine density of neurons in the M1 region [51]. This result was matched by enhanced forelimb strength and motor skills in the tDCS group of mice as well as an increase in synaptic transmission and plasticity at the level of layer II-III connections in the M1 region. At the cellular level, tDCS increased neurotransmitter release and AMPAR/NMDAR ratios, consistent with the increased density of dendritic spines observed in tDCS mice. At the molecular level, the changes in dendritic structure were accompanied by increased levels of BDNF and increased levels of phosphorylation of synaptic plasticity-related proteins including CaMKII, CREB, and NMDAR.

tDCS-induced synaptic plasticity encompasses multiple aspects of neurobiology and neurophysiology, including gene transcription, protein expression, neurotrophic factor regulation, neural signaling, and synaptic remodeling. Given the significant roles of LTP- and LTD-like effects in synaptic plasticity, researchers believe that tDCS is likely to induce long-term changes in brain excitability and activity through LTP- and LTD-like effects. This mechanism contributes to the enhancement of synaptic plasticity and ultimately improves motor performance.

### 2.3. Modulating Neurovascular Coupling to Improve rCBF in the Brain

**Figure 4:**
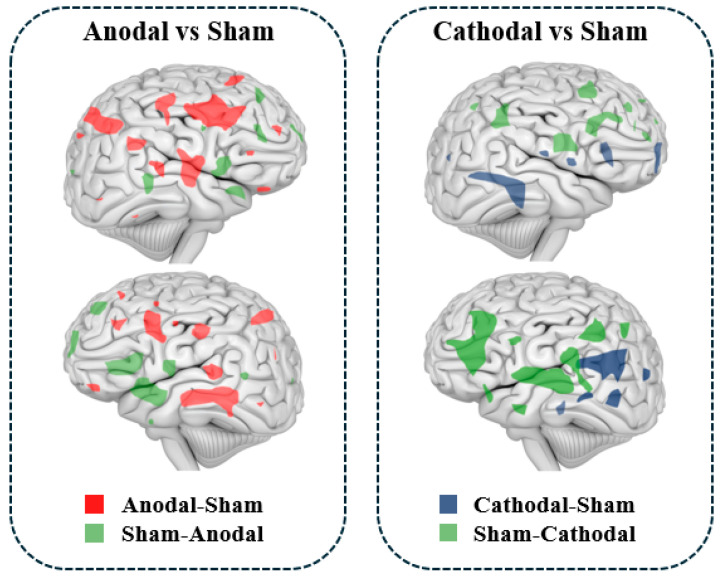
rCBF effects induced by tDCS.

As illustrated in Figure 4, studies indicate that transcranial direct current stimulation (tDCS) can modulate regional cerebral blood flow (rCBF). Anodal tDCS typically leads to a widespread increase in rCBF across the cerebral cortex and subcortical regions, while cathodal tDCS generally results in a decrease in rCBF. Furthermore, changes in rCBF are positively correlated with alterations in cortical excitability induced by tDCS [52,53,54]. Shinde et al. (2021) found that the current density of tDCS was related to finger sequence task performance and that enhanced tDCS current intensity increased rCBF [54]. A similar study found that the current intensity of tDCS affects the distribution of rCBF, and when anodal or cathodal tDCS (current intensity of 0.5 mA, 1 mA, 1.5 mA, and 2 mA) was applied to the left M1 region of the brain, the results showed that the stimulation condition of anodal tDCS both significantly increased the rCBF in the M1 region, and the effect of its stimulation action at a current intensity of 2 mA caused rCBF to significantly increase, while cathodal tDCS consistently decreased rCBF in the M1 region [55]. Finite element modeling revealed that the simulated spatial distribution of rCBF perfusion was highly correlated with stimulation parameters, such as the distribution of the tDCS electric field [55]. In recent years, there has been a gradual increase in the number of cases in which tDCS improves rCBF by modulating local brain activity, thereby improving cognition and brain function. For example, tDCS combined with training significantly improves motor function in stroke patients, and the mechanism may be that the stimulation improves blood flow to the damaged areas of the brain [56,57,58]. In patients with mild cognitive impairment, tDCS significantly improved working memory, attention, and executive function, and this effect was strongly associated with increased rCBF [59]. Several studies have shown that tDCS stimulation resulted in improved mood regulation and symptom reduction in patients with depression, which was associated with increased blood flow to mood-regulating regions of the brain [60,61]. Similar studies have found that tDCS significantly improves memory function in patients with Alzheimer’s disease, suggesting that its potential lies in slowing the process of cognitive decline by promoting the restoration of blood flow to brain regions [62,63,64]. Animal experiments have shown that tDCS can alleviate the dysfunction of neurovascular units in Alzheimer’s disease mouse models, improve blood vessel density and length, and have a certain protective effect on the blood–brain barrier, thus regulating rCBF in the brain [62].

Currently, the neurovascular coupling (NVC) doctrine is considered the predominant explanation for transcranial direct current stimulation (tDCS)-induced changes in regional cerebral blood flow (rCBF). This doctrine emphasizes the complex interactions between neurons and adjacent non-neuronal structures, particularly glial cells and cerebral blood vessels, which together form neurovascular units [65]. According to NVC theory, changes in rCBF induced by tDCS are primarily attributed to the secondary vascular responses of stimulated neurons. Specifically, anodal tDCS enhances vasodilation resulting from neuronal excitation, while cathodal tDCS inhibits vasoconstriction triggered by neuronal stimulation [66]. The immediate changes in rCBF are likely due to the electric field’s influence on the various cellular components of large, medium, and small blood vessels, which first affects the dura mater before concentrating on the cerebrovascular system [66]. However, some studies have reported an instantaneous increase in rCBF under cathodal compared to anodal tDCS, indicating a direct effect of tDCS on vascular tone. The endothelial release of nitric oxide and the production of vasodilatory neuropeptides by astrocytes are believed to be the primary factors responsible for this vasodilatory effect [67,68]. Taken together, tDCS affects changes in rCBF by modulating vasoconstriction or diastole. Increased rCBF provides more oxygen and nutrients to specific areas of the brain and promotes elevated neuronal metabolic activity and function. This enhanced brain function contributes to improved efficiency in motor control, motor learning, and movement execution. In particular, increased rCBF during tDCS stimulation of movement-related brain regions (e.g., motor cortex) may enhance performance in motor planning, movement execution, and motor coordination.

### 2.4. Modulating Brain Network Functional Connectivity to Activate and Strengthen Brain Regions

The brain is a complex network system, with different regions responsible for regulating motor, cognitive, and emotional functions, all of which are closely interconnected [69]. As neuroscience and technology continue to advance, the functional connectivity and synchronization of networks across the cerebral hemispheres have garnered significant interest among researchers. Understanding these aspects of brain networks can enhance our exploration of the brain’s information-processing and expression mechanisms [70,71]. Functional connectivity (FC) refers to the patterns of interactions and information transfer between different brain regions, reflecting the synchronization of neural activity over a specific time period [72,73]. It is measured by analyzing the correlation of neural signals between regions, often employing tools such as functional magnetic resonance imaging (fMRI), electroencephalography (EEG), or magnetoencephalography (MEG). Functional connectivity provides insights into how the brain operates as a cohesive network, particularly during rest or specific tasks [73]. The brain is viewed as a complex system of different functional networks, each associated with a specific cognitive function.

The fMRI technique is a commonly used neuroimaging technique to probe the functional connectivity and synchronization of brain networks [74,75]. It has been found that anodal tDCS applied to the M1 region combined with the fMRI technique significantly increased functional connectivity changes between brain regions, such as sensorimotor, motor, and premotor regions [76]. Anodal tDCS modulated functional connectivity in cortical, cortico-striatal, and thalami-cortical motor pathways [77]. Motor skills are the ability of the human body to complete a target action during movement. Motor-skill learning is the process by which the human body receives various signal stimuli and establishes complex conditioned reflexes under the guidance of the cerebral cortex. It is also the process by which the cerebral cortex establishes a balance between excitation and inhibition [78]. Motor-skill learning adaptations are associated with functional connectivity of brain networks, especially brain regions such as M1, cerebellum, supplementary motor area (SMA), and DLPFC [79]. It has been shown that tDCS increases synaptic plasticity and enhances functional connectivity in premotor, motor and sensorimotor areas [80]. The above mechanisms have positive effects on brain activity in motor-skill learning and promote motor-skill learning. Most studies have shown that tDCS enhances motor skills in healthy adults through activation of different brain regions, such as a decrease in reaction time in a motor-sequence task and an increase in the number of successes in a golf-putting task [81].

**Figure 5:**
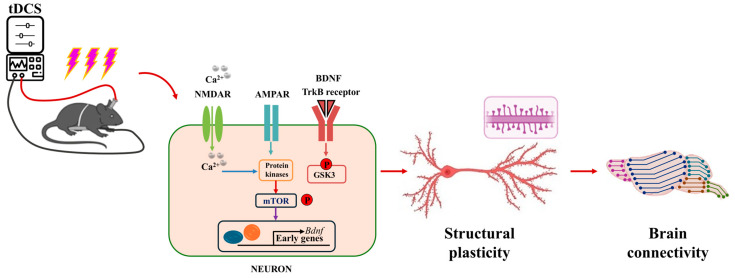
Structural plasticity of neurons by tDCS modulates brain networks.

Polania et al. (2012) found that anodal tDCS of the M1, combined with fMRI results, increased functional connectivity between the M1 region, which controls hand movements on one side, and the premotor and parietal cortical regions that regulate hand movements on the other side. Additionally, increased functional connectivity was observed between the posterior area of the left posterior cingulate cortex and the right dorsolateral prefrontal cortex [76]. Notably, the functional connectivity between the posterior region of the left cingulate gyrus and the right dorsolateral prefrontal cortex was significantly enhanced [76]. Changes in functional connectivity within brain networks can modulate various brain functions, including sleep. Studies have shown that tDCS improves working memory in older adults, with fMRI revealing a significant increase in functional connectivity between the left dorsolateral prefrontal cortex and the left ventral lateral prefrontal cortex in subjects receiving tDCS [82]. Another study investigating the effects of tDCS on sleep in older adults found that high-precision tDCS-induced prolonged sleep duration, while fMRI indicated a reduction in functional connectivity between cortical networks [83]. As research progresses, tDCS is expected to emerge as an alternative therapy for alleviating aging-related issues, sleep disorders, and neurological conditions. Recent findings suggest that brain connectivity is associated with tDCS-induced structural plasticity [84]. As illustrated in Figure 5, tDCS promotes the expression of brain molecules associated with synaptic changes, enhances the LTP-like effect, synaptic transmission, and dendritic spine density, which ultimately improves learning, memory abilities, and functional connectivity within brain networks.

### 2.5. Enhancement of Excitatory Neurotransmitter Levels and Release

Neurotransmitters are a class of specialized chemicals released from the presynaptic membrane during chemical synaptic transmission, serving as messengers to the postsynaptic membrane [85]. Neurotransmitters are released from the presynaptic membrane, pass through the synaptic gap, act on the postsynaptic membrane, and bind specifically to receptors [86]. According to the structure, they are mainly divided into protamine, amino acid, peptide, and other neurotransmitters. The protamine neurotransmitters, such as dopamine and 5-hydroxytryptamine, were the first to be discovered. Peptide neurotransmitters include generators of inhibin, substance P, cholecystokinin, and so on. Amino acid neurotransmitters include glutamate (Glu), glycine, histamine, and others. Other neurotransmitters include arachidonate, nucleotides, etc. Neurotransmitters are essential for maintaining the dynamic homeostatic function of the brain and body. For instance, the balance between excitatory and inhibitory neurotransmitters can influence synaptic plasticity in the brain [87]. Additionally, monoamine neurotransmitters, such as dopamine and serotonin, are implicated in the pathophysiology of various psychiatric disorders.

Studies have shown that the primary mechanism of action of tDCS involves modulating neuronal excitability by influencing the resting membrane potential of neurons [88]. Currently, the changes in neuronal excitability induced by tDCS are categorized into direct, short-term, and long-term effects [89,90], each potentially operating through different mechanisms. The direct effects of tDCS are attributed to alterations in neuronal resting membrane potentials, which depend on the polarity of the electrodes placed on the scalp. The long-term effects are explained by the neuroplasticity model, which posits that current intensity leads to modifications in neuronal synapses, thereby facilitating or inhibiting local neuronal transmission and ultimately enhancing or diminishing synaptic efficacy.

Glu, a key excitatory neurotransmitter, has been extensively studied for its role in synaptic maintenance and plasticity, facilitating learning and memory through alterations in synaptic efficacy. Additionally, changes in Glu concentration are closely related to the modulation of receptors involved in LTP- and LTD-like effects [87]. Magnetic resonance spectroscopy (MRS) is a powerful non-invasive technique that traces signals from hydrogen nuclei in water molecules, allowing for the assessment of various neuronal metabolites, such as N-acetyl aspartate (NAA), creatine (Cr), Glu, glutamine (Gln), gamma-aminobutyric acid (GABA), choline-containing compounds, and inositol [91].

GABA and Glu, as the principal inhibitory and excitatory neurotransmitters in the brain, play vital roles in physiological processes and are closely associated with neurodegenerative diseases [92]. NAA affects neuronal and axonal integrity and serves as a marker of neuronal connectivity [93]. The primary roles of cholinergic compounds and Cr are related to cell membrane integrity and cellular oxidative metabolism, respectively [93]. Inositol, an essential compound in the brain, is involved in biochemical signaling pathways and the synthesis of phospholipids in cell membranes [94]. It acts as an important osmoregulatory substance and astrocyte marker, differentiating between physiological and pathological conditions, and plays a fundamental role in maintaining the brain’s energy status by regulating energy requirements for various cellular functions and activities, including ATP synthesis and the polymerase chain reaction [94].

MRS can reliably identify and quantify brain metabolites in both healthy and clinical populations [91]. In MRS, the signals of individual molecules are quantified and separated based on their frequencies and molecular characteristics, allowing for accurate measurement of molecular concentrations through frequency analysis and optimization of the signal-to-noise ratio. Recently, a multimodal study demonstrated that Glu concentrations were elevated in the region stimulated by tDCS [95]. In cases of autism spectrum disorders, a significant increase in brain metabolites such as NAA, Cr, and myo-inositol concentrations was observed following anodal tDCS [96]. Another study investigating the long-term effects of anodal tDCS on the primary motor cortex (M1) showed an association with glutamatergic plasticity [97]. Furthermore, anodal tDCS applied to the left primary sensorimotor area significantly increased Glu levels in the precentral gyrus and enhanced functional connectivity in this region [98].

**Figure 6:**
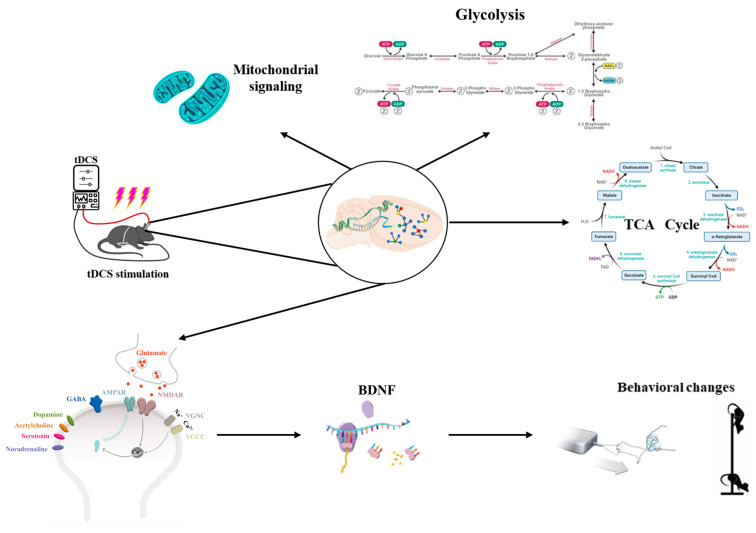
Analysis of the effects of tDCS on metabolic pathways.

Research has indicated that metabolite levels, particularly Glu, change during or after tDCS. Specifically, the concentration of excitatory neurotransmitters, such as Glu, significantly increases following tDCS intervention in cortical regions of the brain [99,100]. As shown in Figure 6, tDCS can alter the metabolic profile of brain tissue, impacting bioenergy-related metabolic pathways such as glycolysis and mitochondrial function [101]. Additionally, tDCS influences calcium signaling and metabolism by regulating processes related to energy production. Theoretical modeling and animal studies have demonstrated that repetitive transcranial magnetic stimulation can modify neurotransmitter concentrations in cortical and subcortical regions, with low-intensity repetitive transcranial magnetic stimulation inducing increases in concentrations of neurotransmitters such as dopamine (DA), Glu, homovanillic acid (HVA), dihydroxyphenyl acetic acid (DOPAC), serotonin (5-HT), and 5-hydroxyindoleacetic acid (5-HIAA) in the cerebral cortex of mice [102]. As an important excitatory neurotransmitter in the central nervous system, Glu plays a key regulatory role in mediating synaptic plasticity and neuronal excitatory transmission, and DA is important for fine motor regulation [103]. Glu released from the presynaptic membrane reacts with Glu ionotropic and metabotropic receptors in the postsynaptic membrane to mediate synaptic plasticity and enhance the LTP-like effect. tDCS stimulation induces an increase in Glu content, which regulates NMDAR and AMPAR sites, triggers calcium inward flow, enhances the efficiency of synaptic transmission, and promotes the expression of BDNF, while an increase in DA content facilitates the mastery of fine motor skills. The increase in DA content is conducive to the mastery of fine motor skills and the enhancement of motor performance. However, it remains unclear whether findings from motor cortex experiments can be generalized to other critical regions, such as the dorsolateral prefrontal cortex. Therefore, further studies are required to explore the relevance of these results to other brain regions.

## 3. Summary and Outlook

tDCS holds significant promise as a tool for neuroscience research due to its non-invasive, efficient, user-friendly, cost-effective, and portable nature. This article reviews the current effects of tDCS on neuronal, synaptic, and brain network levels and discusses the neurophysiological mechanisms through which tDCS enhances athletic performance. These mechanisms include regulating neuronal resting membrane potential to alter cortical excitability, enhancing synaptic plasticity to modulate the LTP-like effect, improving rCBF through neurovascular coupling, modulating the functional connectivity of brain networks to activate and strengthen specific brain regions, and increasing neurotransmitter levels and release to regulate brain function. Future research should focus on further elucidating the specific neurophysiological mechanisms by which tDCS enhances human athletic performance and expanding its applications to other fields, such as cognitive enhancement, applied psychology, and brain network functional connectivity.

## Data Availability

The data presented in this study are available on reasonable request from the corresponding author.
